# *Corynebacterium striatum* meningitis combined with suspected brain and lung abscesses: a case report and review

**DOI:** 10.1186/s12879-020-05114-3

**Published:** 2020-06-01

**Authors:** Ming-Jie Zhang, Xiao-Jie Cao, Jin Fan, Ze-Gang Yin, Ke Yu

**Affiliations:** Department of Neurology, The General Hospital of Western Theater Command, No.270 Rongdu Avenue, Jinniu District, Chengdu, 610083 Sichuan Province People’s Republic of China

**Keywords:** *Corynebacterium striatum*, Meningitis, Abscess, Case report

## Abstract

**Background:**

Intracranial infections with *Corynebacterium striatum* (*C. striatum*) have been described sporadically in the literature over the last two decades. However, *C. striatum* meningitis combined with multiple abscesses has not been published before.

**Case presentation:**

In this report, we describe the clinical and imaging findings in a 54-year-old woman with meningitis caused by *C. striatum* and combined with suspected brain and lung abscesses. This patient who underwent multiple fractures and a recent cut presented with headache and paraphasia. *C. striatum* was isolated in cerebrospinal fluid and supposedly transmitted from the skin purulent wound through blood. The patient was treated with intravenous vancomycin and had a transient improvement, but died finally. Multiple abscesses, especially in the brain, could be a reason to explain her conditions were deteriorating rapidly.

**Conclusions:**

Note that *C. striatum* can cause life-threatening infections. Early identification and diagnosis, early administration of antibiotics to which the bacterium is susceptible, and treatment of complications will be beneficial in patients with *C. striatum*-related infection.

## Background

*Corynebacterium* species are part of the resident skin flora. Among these, *Corynebacterium striatum* (*C. striatum*) is a kind of causative bacteria to induce various types of infections mainly including lower respiratory tract infection, isolated bacteremia and central line infections [1]. However, only a few reports have mentioned meningitis caused by *C. striatum* over the last two decades [2–5]. Furthermore, *C. striatum* meningitis combined with multiple abscesses has not been published before. In this report, we describe one patient with meningitis caused by *C. striatum* and combined with suspected brain and lung abscesses.

## Case presentation

A 54-year-old woman presented to the neurology department of our hospital on 29 October 2019 complaining of headache, blurred vision, vomiting and paraphasia. This patient had had rheumatoid arthritis and osteoporosis, and taken the drugs irregularly over the past decade. She broke her right femur 5 years ago and right leg 2 years ago. She underwent fracture repair surgeries successively and sustained the plate internal fixation in her right leg. The surgical wounds had healed without obvious infection. Accidentally, she cut her left leg over 40 days ago and smeared Chinese herbal ointment to promote healing. However, the treatment effect was poor and she had not paid enough attention to protect against infections. Ultimately, she left an unhealed and purulent wound visibly. Family and psycho-social history including relevant genetic information was normal.

At the time of admission, the patient’s vital signs were normal without fever. She was confused and had simple language reaction to painful stimuli. She did not cooperate to complete neurological examination which showed a stiff neck and positive right pathological sign.

Laboratory tests of blood taken at admission showed a white blood cell (WBC) count of 14.61 × 10^9^/L with 84.5% neutrophils. High-sensitivity C-reactive protein was 256.59 mg/L and procalcitonin was 4.89 ng/mL, both far above normal values. Lumbar puncture was then performed after excluding contraindications, in especial brain shift on computed tomography (CT) plain scanning. A lumbar puncture yielded cloudy cerebrospinal fluid (CSF) with initial pressure over 400mmH_2_O. CSF analysis revealed an elevated total nucleated cell count of 1210 × 10^6^/L (90% multiple nuclear cells), an increased protein concentration of 1.58 g/L (normal value, 0.01 ~ 0.45 g/L) and a decreased glucose concentration of 0.39 mmol/L (blood glucose 5.44 mmol/L). Considering the available information, we empirically made a diagnosis of purulent meningitis and immediately treated with intravenous vancomycin (1 g every 12 h) and meropenem (1 g every 8 h) to cover all common causative microorganisms until culture results were known.

**Fig. 1:**
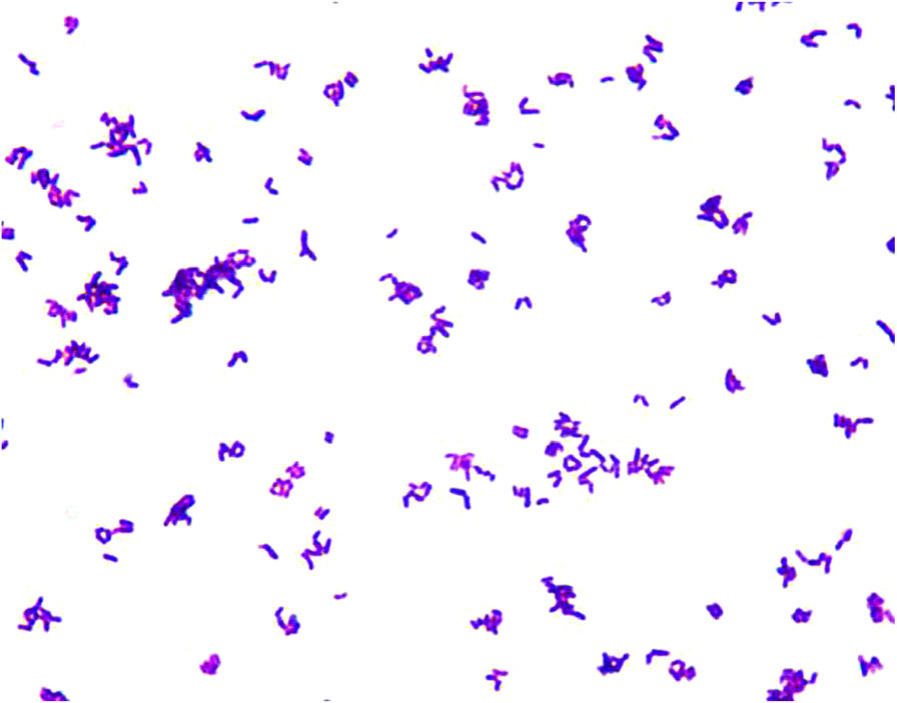
Gram stain of *C. striatum* shows typical coryneform morphology

After a 24-h incubation period, gram staining of the cloudy CSF demonstrated the presence of gram-positive bacteria (Fig. 1). After 72 h of treatment, the patient had a marked improvement in health conditions and laboratory tests. She became alert, correct-replied and well-cooperated. In the meantime, her headache gradually relieved. Blood WBC count, high-sensitivity C-reactive protein and procalcitonin were all decreased. Total nucleated cell count of CSF for reexamination decreased to 110 × 10^6^/L, and CSF culture yielded no bacterial growth. Five days later, the first bacterial culture was subsequently identified as *Corynebacterium striatum* (*C. striatum*). Susceptibility testing was not performed by our laboratory department due to a lack of drug sensitivity guidelines for this kind of bacteria in our country. Intravenous vancomycin and meropenem therapy was continued. Blood culture taken before the start of antibiotic therapy remained negative. The CSF and blood levels of vancomycin were not measured.

**Fig. 2:**
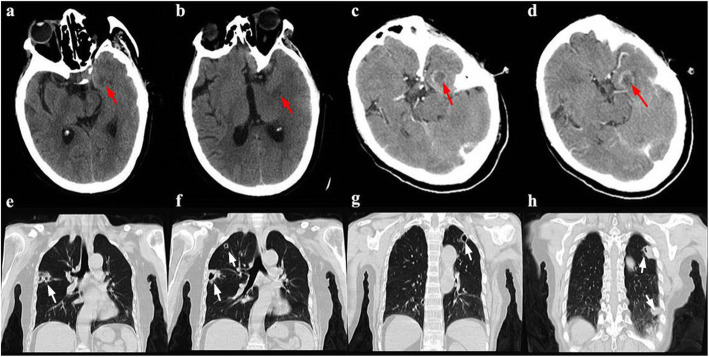
Head (**a**-**d**) and chest (**e**-**h**) CT. CT plain scanning of the cranium (**a**-**b**, red arrow) shows a hypodense lesion in the medial temporal region. CT scanning with contrast enhancement (**c**-**d**, red arrow) shows the lesion characterized by a hypodense center and a hyperdense ring that is considered to be a suspected brain abscess. Chest CT scanning (**e**-**h**, white arrow) shows multiple and scattered cavitary lesions in peripheral lungs

However, head and chest CT with contrast enhancement examinations (unable to complete magnetic resonance imaging, MRI) showed intracranial and pulmonary abnormal lesions, which were considered as abscesses (Fig. 2). After 9 days from admission, her conditions were deteriorating rapidly with coma and anisocoria. Dehydration therapy to decrease intracranial pressure was used immediately and the patient progressed smoothly to complete additional tests. A head CT was rearranged promptly and detected that the previous lesion was enlarged questionably. Considering that no definite brain shift was shown, we performed the lumbar puncture again to confirm CSF changes on the premise of the patient’s conditions and her family’s full consent. We followed the operating guidelines to reduce and slow the release of CSF. The lumbar puncture revealed cloudy CSF with initial pressure of 350mmH_2_O, an increased total nucleated cell count of 536 × 10^6^/L and protein concentration of 1.40 g/L. In the next day, this patient had respiratory and circulatory failure gradually. In spite of giving cardio-pulmonary resuscitation and mechanical ventilation, this patient eventually died. As far as available information was concerned, we speculated that the intracranial abscess ruptured and formed cerebral hernia. In the course of the hospitalization, the patient’s family firmly refused any surgical operations because of the costs and risks.

## Discussion and conclusion

*C. striatum* has been established as an emerging multidrug-resistant and opportunistic pathogen, which was traditionally known as a common component of the normal microbiota of skin and mucosal surfaces [1]. *C. striatum* was commonly isolated from wounds, respiratory specimens, tissue specimens, bone specimens and blood cultures. Therefore, most cases of *C. striatum* infection manifested as pneumonia, bacteremia/septicemia, endocarditis and peritonitis. And the scope of these infections has been broadening.

In the present case, *C. striatum* was isolated from CSF and identified as the causative pathogen. To our knowledge, only a few previous reports have implicated *C. striatum* in central nervous system infection. The first proven case of meningitis caused by *C. striatum* was published in 1996, which was a 23-year-old Canadian man with a direct communication between the skin and the CSF through a catheter left after an extensive nerve reconstructive surgery [2]. Subsequently, the report from United Kingdom showed that the cultures of CSF samples yielded *C. striatum* from three children at the ages of 13-month, 20-month and 6-year, respectively [3]. These cases were all treated with a ventriculoperitoneal (VP) shunt and the cultures of VP shunt samples also revealed *C. striatum*. Two Turkish cases of meningitis due to *C. striatum* both underwent an external ventricular drainage after neurosurgery operations [4]. Additionally, a French man suffered a severe trauma leading to multiple craniocerebral injuries and skull fractures and subsequently had the *C. striatum* meningitis [5]. However, *C. striatum* meningitis combined with multiple abscesses has not been published before. Bacteria can enter the brain to induce intracranial infection through contiguous spread, hematogenous dissemination or with other unknown mechanisms [6]. After analyzing available literatures published, we discover the fact that there seems to be a direct communication between the skin and the CSF through the skin wound, bone fracture or drainage catheter to explain how *C. striatum* invades to CSF and causes meningitis. In the present case, it seemed that there was no direct communication between the skin and the CSF, in spite of those extracranial bone fractures. Hence, it suggested hematogenous spread of bacteria from the skin purulent wound was the primary cause of meningitis and forming abscess.

Cranial imaging should be performed in patients with intracranial infection, especially suspected brain abscess. CT scanning with contrast enhancement provides a rapid means to detect abscesses, but lacks sensitivity and specificity. Head CT scanning with contrast enhancement of the present patient showed the lesion characterized by a hypodense center and a hyperdense ring that was considered to be a suspected brain abscess. Comparatively, MRI combined with diffusion-weighted and apparent-diffusion coefficient images is a more valuable diagnostic tool [6]. Unfortunately, the present patient was unable to complete MRI because of the surgical plate in the body. The bacteria positive rate of CSF cultures in patients with brain abscess was not considerable, unless with coexisting meningitis [7]. Finally, the causative microorganism, *C. striatum*, in the present case was identified from CSF culture. However, the association between the *C. striatum* and abnormal lesion in brain was not definitely clear without evidence from biopsy or drainage culture. Additionally, pulmonary cavitary lesions on CT were detected in the present patient and abscesses could not be ruled out as well. The most common causes of lung abscess are aspiration pneumonia, transdiaphragmatic spread, and hematogenous seeding commonly associated with right-sided endocarditis or septic thrombophlebitis [8]. These cavitary lesions were multiple and scattered in peripheral lungs, which was more supportive of hematogenous spread of bacteria. Unfortunately, blood and sputum cultures were negative.

*C. striatum* is relatively resistant to many of the commonly used antibiotics with Gram-positive activity. It was demonstrated that vancomycin, linezolid and daptomycin were generally classified as active antibiotics [1]. However, the failure of therapy with daptomycin in some case reports was associated with the rapid emergence of isolates with a resistant phenotype [1]. Vancomycin was found to be the universally used antibiotic in cases with *C. striatum* meningitis published in the above-mentioned studies and the present case report. Note that the different treatment effects and clinical outcomes were also attributed to severity of infection and patients’ clinical manifestations. The fact that the present patient had coma, anisocoria, respiratory and circulatory failure associated with the intracranial infection could explain why she died. Multiple abscesses, especially in the brain, could be a reason that her conditions were deteriorating rapidly.

As a conclusion, these published cases confirm that *C. striatum* has the potential to cause meningitis especially in patients with direct communication between the skin and the CSF. No denying that hematogenous spread of *C. striatum* is also worthy of attention. The frequency of *C. striatum* infections appears to be increasing along with the improvement of feasibility and accuracy of the identification from clinical cultures. Note that *C. striatum* can cause life-threatening infections. Early identification and diagnosis, early administration of antibiotics to which the bacterium is susceptible, and treatment of complications will be beneficial in patients with *C. striatum*-related infection.

## Data Availability

All data generated during this study are included in this published article.

## Abbreviations

*C. striatum*: *Corynebacterium striatum*
CSF: Cerebrospinal fluid
CT: Computed tomography
WBC: White blood cell
